# Genomic characterization and pre-clinical evaluation of a new polyvalent lytic *Loughborough* phage

**DOI:** 10.1007/s00253-025-13559-2

**Published:** 2025-08-02

**Authors:** Mahmoud M. Sherif, Neveen A. Abdelaziz, Sarra E. Saleh, Khaled M. Aboshanab

**Affiliations:** 1 Department of Microbiology and Immunology, Faculty of Pharmacy, Sixth of October City, Ahram Canadian University, Giza, 12451 Egypt; 2 Department of Microbiology and Immunology, Faculty of Pharmacy, Ain Shams University, Cairo, 11566 Egypt

**Keywords:** CRAB, *Acinetobacter baumannii*, MDR *Salmonella*, Polyvalent phage, Molecular analysisSalmonella_, Hydrogel, Thermal animal model

## Abstract

**Abstract:**

Carbapenem-resistant *Acinetobacter baumannii* (CRAB) has become a critical concern that necessitates the development of novel antimicrobial approaches. One of the most promising innovative approaches for combating CRAB infections is using effective and lytic bacteriophages (phages), known as phage therapy. Therefore, we recovered and characterized a polyvalent lytic Salmonella_phage_VB_ST-SA173, producing lytic activity against 6 CRAB clinical isolates and 3 multidrug-resistant (MDR) *Salmonella* serovars. Throughout pH 2–10, and thermal stability at up to 60 °C, the phage maintained its stability and lytic activity against the tested isolates. The presence of a tailed phage with a characteristic prolate head and a contractile tail was detected by the transmission electron microscope (TEM). According to the Oxford nanopore sequencing data, the genome of Salmonella_phage_VB_ST-SA173 was 53,636 bp in size, contained 45.9% G + C, and had 53 opening reading frames (ORFs). According to the TEM, ORFs, and BLASTn analysis findings, it was proved that the Salmonella_phage_VB_ST-SA173 belongs to the *Loughboroughvirus* genus. The efficacy of the phage-formulated Carbopol 940 hydrogel in wound healing was assessed preclinically in an infected burn wound animal model with a CRABa clinical isolate. The survival rate was enhanced in the phage-treated group compared to the untreated control groups. Histopathological analysis showed improved wound healing in the form of apparently healthy skin with apparently normal epidermal and dermis layers. In conclusion, depending on its in vitro and physicochemical traits, the phage-loaded hydrogel is expected to be a promising candidate for clinical trials against human CRAB-related skin infections.

**Key points:**

•* A polyvalent Loughboroughvirus phage showed lytic activity against CRAB and Salmonella serovars.*

•* The phage showed stability at a wide range of pH and temperature.*

•* The phage hydrogel enhanced healing in the burn-wound animal model infected with CRABa.*

**Supplementary Information:**

The online version contains supplementary material available at 10.1007/s00253-025-13559-2.

## Introduction

According to the World Health Organization (WHO), antimicrobial resistance (AMR) is one of the most serious risks to global health, accounting for 1.27 million deaths globally in 2019 (WHO 2023). If no alternative intervention measures are implemented, the Centers for Disease Control (CDC) predicts that 10 million mortalities will occur annually by 2050 (CDC 2019). *A. baumannii* is a member of ESKAPE pathogens that possess potential mechanisms of antibiotic resistance and are accountable for the majority of nosocomial infections worldwide (Santajit and Indrawattana 2016).

*A. baumannii* is a ubiquitous aerobic Gram-negative coccobacillus that is typically isolated from both natural and medical environments and is a catalase-positive and oxidase-negative organism (Wang et al. 2024). A wide range of opportunistic hospital-acquired and life-threatening infections were attributed to *A. baumannii* pathogen (Lee et al. 2017). *A. baumannii* is one of the most resistant bacteria on the planet due to its plastic genome, virulence factors, and endless resistance mechanisms (Chakravarty 2020). Unfortunately, resistance to carbapenems, regarded as the most effective antibiotic against MDR *A. baumannii* infections, has been reported (Abdelaziz et al. 2022). In *A. baumannii* isolates, researchers have detected all four classes of β-lactamase enzymes, which are the primary mechanism of resistance to carbapenems (Chakravarty 2020). Based on the above, the CDC and the WHO have ranked CRAB as an urgent concern that necessitates discovering novel antimicrobial approaches (CDC 2019) (WHO 2017).

Phage therapy, which has regained great attention over the last decade, is regarded as one of the auspicious solutions to the AMR crisis (Ikpe et al. 2024). Phages comprise the planet’s most prevalent biological entity, with an approximate 10^31^–10^32^ phages in different ecosystems (Lin et al. 2017). After attachment to bacterial cells during infection, phages inject their genetic material into the bacterial cell. After that, phages typically go through one of two life cycles: lytic or lysogenic (Brives and Pourraz 2020). In the lytic cycle, virulent phages use the bacterial replication machinery to create the phage progeny of the next generation, causing the hydrolysis of the bacterial peptidoglycan (Lin et al. 2017). Inversely, the lysogenic phages remain latent in the bacterial host and carry out DNA replication without destroying the host bacterial cells (Wei et al. 2020). Consequently, only lytic phages can be applied for phage therapy. Phage therapy has numerous benefits, including autonomous dosing, minimal intrinsic toxicity, low destructive effect on normal flora, reduced potential for resistance induction, and easy and rapid discovery with low cost. However, a narrow host range where the phages are limited to a few strains, species, or, in rare cases, bacterial genera, is one of the main limitations of phage therapy (Hyman and Abedon 2010).

Phages with a wide host range are frequently isolated from bacterially diverse environments, like wastewater or free-range dairy farms. This diversity supports genetic exchanges that increase the possibility of phage recombination, enabling the phages to target a wider range of hosts (Gencay et al. 2019). Polyvalent phages can infect bacterial cells from at least two different genera (Gambino et al. 2020). Polyvalent phages can infect hosts from different genera that have a similar conserved receptor. Nevertheless, despite the diversity of phages on Earth, only a few isolated phages are effective against *A. baumannii* isolates (Tu et al. 2023). Consequently, phage therapy requires the isolation and characterization of more potent phages against MDR *A. baumannii* isolates (Tan et al. 2023). In this study, we evaluated the lytic efficacy of a recently identified polyvalent Salmonella_phage_VB_ST-SA173 lysate against CRAB and *Salmonella* spp. clinical isolates. Furthermore, the phage-formulated hydrogel was preclinically evaluated in an infected burn wound animal model with CRAB isolate. The genome of the isolated phage was entirely sequenced and annotated, and it was morphologically analyzed.

## Materials and methods

### Identification of CRAB and MDR *Salmonella* spp. clinical isolates and evaluation of susceptibility to antibiotics

For the isolation of phages from sewage samples, 28 CRAB clinical isolates were used. The isolates were previously identified in our lab, where the isolates’ susceptibilities and molecular detection of *β-*lactamase-encoding genes were carried out (Sherif et al. 2021). The microbiology lab at Ain Shams University, Faculty of Medicine, El-Demerdash Hospital in Cairo, Egypt, provided all the clinical isolates. Using *A. baumannii* ATCC 19606 as a positive control, the isolates were identified by using standard PCR to detect the *bla*_OXA 51-like_ gene, which is intrinsic to *A. baumannii* (El Far et al. 2019). Utilizing the micro-broth serial dilution method according to the CLSI guidelines, the clinical isolates’ minimum inhibitory concentrations (MICs) were verified with five different antibiotics with distinct mechanisms of action: colistin, imipenem, doxycycline, levofloxacin, and amikacin. The reference strain used in this method was *E. coli* ATCC 25922. Furthermore, five MDR *Salmonella* spp. clinical isolates (coded strain S. Typhimurium strains 1 & 2, and 3, S. Paratyphi A strain 28, S. Paratyphi C strain 29) that were identified and susceptibility described in a previous study in our lab (Youssef et al. 2024) were selected to evaluate the host range of the phage.

According to earlier descriptions, the isolates were categorized as MDR (Girija As and Priyadharsini 2019). The 28 CRAB and MDR *Salmonella* spp. clinical isolates were deposited in the culture collection Ain Shams University (CCASU; https://ccinfo.wdcm.org/collection/by_id/1186).

### Recovery and spot test of phages

Active phages were isolated via the enrichment method using wastewater samples from urban and medical sources (Abdelaziz et al. 2023). The procedure involved adding 20 mL of a clear sewage sample to an equal volume of 2 × nutrient broth medium that contained 10 mg of CaCl_2_. The mixture was then supplemented with 1 mL of 24-h bacterial host cultures (0.5 McFarland). After 48 h of shaking at 37 °C, the mixture was centrifuged for 10 min at 4000 × g, filtered through a 0.22-µm cellulose acetate sterile syringe filter, and then kept at 4 °C. Ultimately, a spot test was used to determine whether active lytic phages were present in the freshly collected lysates (Lin et al. 2010). For the spot test, 100 µL of the bacterial broth in its exponential growth phase was added to 4 mL of molten soft agar (0.7%), which was then immediately placed on standard nutrient agar plates (1.5%). Ten microliters of the phage lysate were spotted on the top layer of the agar after it had solidified. After 24 h of upright incubation at 37 °C, the plates were examined for the development of a clear zone on bacterial lawns.

### Plaque formation assay, purification, and propagation

The double-layer overlay agar assay was applied to advance the phage lysate that produced a consistent positive result with the spot test to the quantitative plaque assay (Abo Kamer et al. 2022). In brief, 100 µL of bacterial suspension (10^8^ CFU/mL) was blended with 100 µL of each dilution of the phage lysate, which had been tenfold serially diluted in phosphate buffer saline, and the mixture was left at room temperature for 10 min. After that, 4 mL of molten soft agar (0.7%) was mixed with this mixture and then promptly transferred onto standard nutrient agar plates (1.5%) which were incubated at 37 °C for 24 h.

A sterile micropipette tip was used to touch a single plaque, which was then inoculated into 2 mL of sterile nutrient broth (2 ×), kept for 2 h at 37 °C, and purified in five successive steps using the double-layer overlay agar assay (Merabishvili et al. 2014). For propagation, the phage isolation process was repeated three times using phage lysate instead of a sewage sample (Youssef et al. 2024). The high titer of phage lysate was filtered through a 0.22-µm sterile syringe filter and stored at 4 °C with an equal volume of sterile glycerol at − 80 °C (Abo Kamer et al. 2022).

The following equation was used to estimate the phage titer in terms of plaque-forming units (PFU) per mL (PFU/mL) (Abd-Allah et al. 2022).

1 $$PFU/mL=number\;of\;plaques/\left(volume\;of\;plague\;plated\left(mL\right)\;\times\;\;dilution\right)$$

### Phage host range

In order to determine the host range of the phage lysate that produced the clearest zone on bacterial lawns with spot test, the lytic activity of the phage lysate was evaluated against the remaining 27 CRAB clinical isolates and 5 MDR *Salmonella* spp. clinical isolates.

### TEM imaging

TEM was used to examine the morphological features of the selected phage lysate. To put it briefly, 2% (w/v) phosphotungstic acid (pH 7.2) was used to negatively stain 10 µL of pure high titer phage lysate (about 10^10^ PFU/mL) that had been adsorbed on a carbon-coated 200 mesh copper grid (Kalatzis et al. 2016). Following that, phage visualization was carried out at the National Research Center (Cairo, Egypt) using a TEM (JEM-2100, HRTEM, JEOL, Japan).

### pH and thermal stability

The stability of the phage was assessed throughout a wide pH range (1–12). One milliliter of phage lysate was mixed with 1 mL of nutrient broth (2 ×), which had been previously adjusted with either 1 M sodium hydroxide (NaOH) or 1 M hydrogen chloride (HCl) to a particular pH. Using the spot test, phage infectivity was assessed at various pH values following a 1-h incubation period at room temperature (Mahmoud et al. 2021). Additionally, for 1 h, the phage stability was observed in a water path that had been previously modified at various temperatures (40, 50, 60, 70, 80, and 90 °C). An aliquot was then aspirated and examined for maintenance of lytic activity (Abd-Allah et al. 2022). Experiments on temperature and pH stability were carried out in triplicate.

## Molecular analysis of the phage

### Phage DNA isolation and library preparation

Following the manufacturer’s instructions, a phage DNA isolation kit (cat. no. 46800, Norgen Biotek Corp., Canada) was used to extract DNA from a purified high titer of phage lysate (about 10^10^ PFU/mL). The Qubit 4 Fluorometer (Thermo Fisher Scientific, Waltham, MA, USA) was used to quantify isolated DNA.

Following the manufacturer’s instructions, the Rapid Sequencing Kit (SQK-RAD004, Oxford Nanopore Technologies, Oxford, UK) was used to prepare the library.

### Oxford nanopore sequencing and assembly

The generated library was loaded onto certified R9.4.1 flow cells (FLO‐MIN106, Oxford Nanopore Technologies) in order to conduct the molecular sequencing. Data was collected using Oxford Nanopore Technologies’ MinKNOW software, version 23.11.5. The MinION™ sequence readings were converted into fastq files using the Dorado basecall server version 7.3.9 (Oxford Nanopore Technologies) on AWS EC2 g4dn.xlarge. Fastq files were categorized using Kraken2 and visualized using Recentrfuge. Flye was used for the de novo assembly, while Medaka was used to refine the draft assembly three times. The PATRIC BRC and RAST algorithms were used to examine the final consensus sequence (Davis et al. 2020) (Parrello et al. 2021). After being annotated, the phage genome’s final assembled sequence was submitted to the NCBI GenBank database with the accession code PQ351918.

### Formulation of topical phage-loaded hydrogel

For the purpose of Carbopol 940 hydrogel (1%) formulation, 1 g of Carbopol powder was dissolved in 100 mL of distilled water and then mixed with a magnetic stirrer at room temperature for 1 h at 8000 rpm. Triethanolamine was used to jellify the polymer and adjust the pH to 7 ± 0.2 (Algin Yapar et al. 2020). After that, 9 mL of hydrogel was put into glass vials and autoclaved for 15 min at 121 °C for sterilization (Ferreira et al. 2023). The ultimate titer (about 10^8^ PFU/mL) was then obtained by aseptically adding 1 mL of phage lysate with the initial titer (roughly 10^9^ PFU/mL) to the 9 mL of sterilized hydrogel vials (Yan et al. 2021). Before use, the phage-loaded hydrogel was kept at 4 °C.

### In vitro antibacterial activity and stability study

The antibacterial activity of the propagated phage lysates alone (positive control), tested hydrogels, and control hydrogel (negative control) was qualitatively determined by spot test (Dehari et al. 2023). In brief, 10 µL from different samples was dropped on the bacterial lawns (0.5 McFarland) on the nutrient agar plate, incubated at 37 °C for 24 h, then evaluated for clear zone formation on bacterial lawns. For stability assessment, the phage-loaded hydrogel was refrigerated at 4 °C for 1 month, then evaluated for lytic activity maintenance on bacterial lawns via spot test.

## Preclinical evaluation of topical phage-loaded hydrogel

### Animal study

Throughout the experiment, 28 mature male Wistar albino rats weighing between 200 and 220 g were employed. All rats were housed in open cages, provided with food devoid of antibiotics, and offered free access to water. Air conditioning was employed to maintain a steady temperature of 25 °C in their quarters. The research ethical committee of Ain Shams University’s Faculty of Pharmacy in Egypt reviewed and approved the entire study (Protocol approval number: ACUC-FP-ASU-RHDIRB2020110301-REC#206).

### Experimental animal wound model

After the shaving of the burn site, a preheated rectangular metal bar measuring 1 × 2 cm and 1 mm in thickness was used to induce burns on the dorsal side. Next, 0.5 mL of bacterial suspension (10^8^ CFU/mL) was applied to the burn wound site to provoke burn wound infection with CRABa clinical isolate (Kusradze et al. 2016). Six control groups and one test group, four rats each, were applied as described before (Mabrouk et al. 2022).

### Treatment

On the infected burn, the tested hydrogel, control hydrogel, Garamycin®, and Mebo® were applied topically 2 h after infection. For 14 days, the therapy was administered twice daily. Three days after infection, the animals’ survival rate was recorded. Dead animals were removed from the groups and were only taken into account when estimating death rates. Following intraperitoneal anesthesia with a mixture of 60 mg/kg ketamine and 10 mg/kg xylazine, the rats were cervically dislocated to end their lives. Following the animals’ sacrifice, the dorsal skin at the wound site was promptly removed and preserved in 10% formalin solution for histological analysis (Sakr et al. 2021).

### Histopathological examination

Skin samples were fixed in 10% neutral buffer formalin for 72 h, decalcified in 10% formic acid, trimmed, cleaned in water, dehydrated in increasing grades of ethyl alcohol, clarified in xylene, and embedded in paraffin for histological analysis. A rotatory microtome was used to create 5-µn-thick tissue sections at the central zones of different wound samples to display the various layers of skin. Hematoxylin and eosin (H&E) staining was applied according to standard protocols on tissue cuts, and tissues were then blindly inspected for histological alterations (Mabrouk et al. 2022).

### Statistical analysis

Experiments were conducted in triplicates, and the observed results were determined by taking the computed mathematical mean.

## Results

### Recovery and screening of phage activity against CRAB clinical isolates

**Fig. 1:**
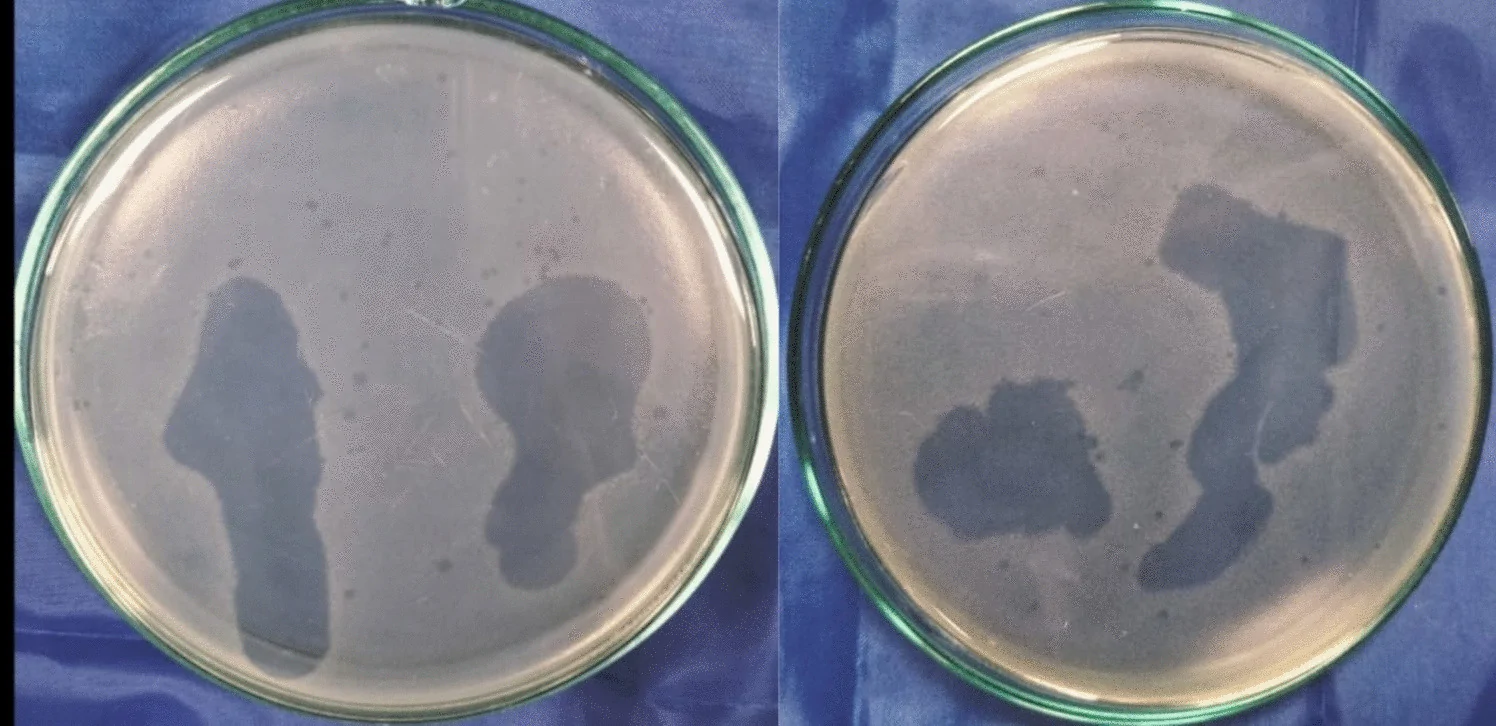
Phage lysate spot test that exhibited the most distinct lytic zones on bacterial lawns

**Fig. 2:**
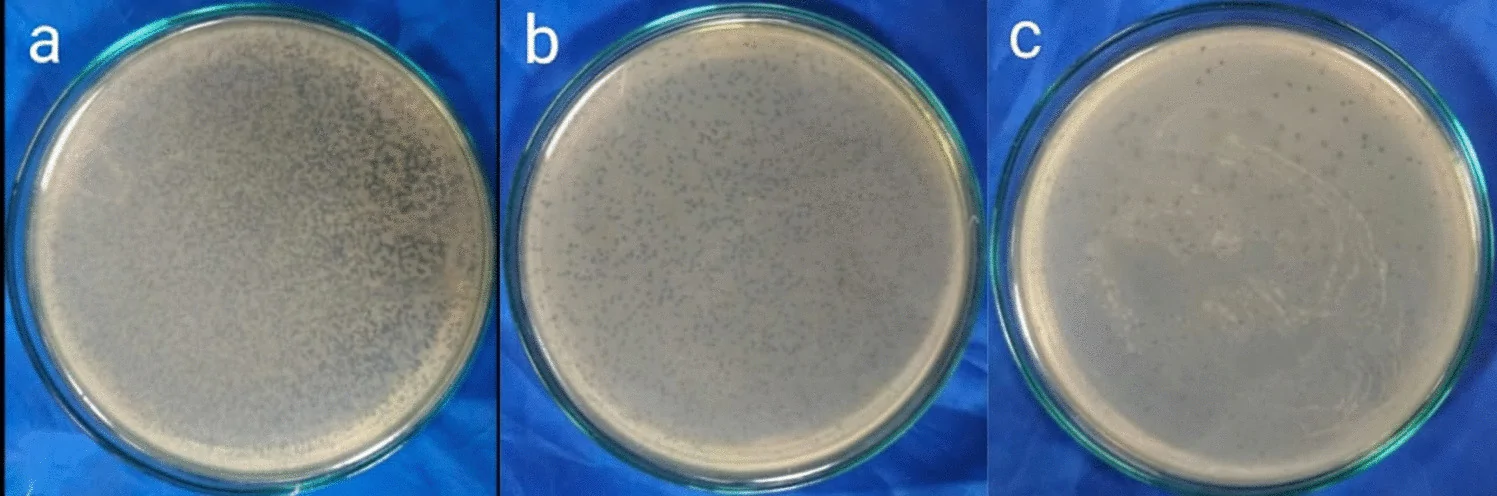
Results of the plaque assay. **a** A sheet of interrupted bacterial growth (net-shaped) was observed at dilutions (10^−3^, 10^−4^); **b** too numerous to be counted (TNTC) plaques were observed at dilutions (10^−5^, 10^−6^, 10^−7^); **c** well-defined, circular plaques were observed at dilution (10.^−8^)

Sewage samples were used to isolate several phage lysates. The lytic activity of the separated lysates against CRAB clinical isolates was assessed. For further investigation, the phage lysate that exhibited the most pronounced inhibitory zone on bacterial lawns and consistently positive spot test was selected (Fig. 1). Using the double-layer overlay agar assay technique (Fig. 2), the chosen phage lysate exhibited a consistently high initial titer (about 10^10^ PFU/mL), which was estimated using (Eq. 1) was selected. The growth of the bacterial host was suppressed by the dilutions (10^−1^, 10^−2^). The isolated phage was deposited in the CCASU (https://ccinfo.wdcm.org/collection/by_id/1186) culture collection under the code Salmonella_phage_VB_ST-SA173.

### Host range

In addition to the host strain coded CRABa, the isolated phage lysate exhibited distinct lytic spots against five additional CRABb, c, d, e, and f clinical isolates (21.43%). Table S1 displays the carbapenemase genes and antibiogram of the CRAB host isolates (CRABa–f). Interestingly, phage lysate also showed lytic zones against 3 MDR *Salmonella* spp. clinical isolates, including *S. Typhimurium* strain 2, *S. Paratyphi* A strain 28, and *S. Paratyphi* C strain 29.

### Phage imaging with TEM

**Fig. 3:**
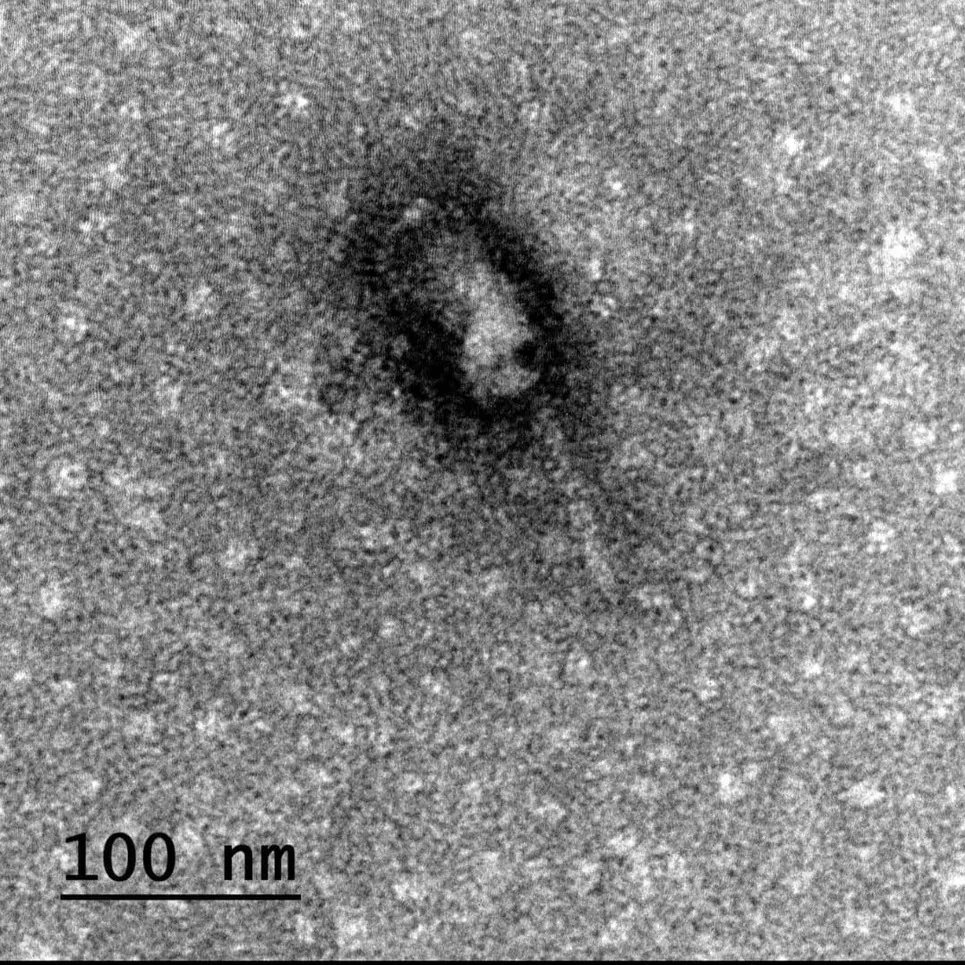
TEM showing a tailed phage with a characteristic prolate head and a contractile tail (myoviral morphotype)

As depicted in (Fig. 3), a tailed phage with a characteristic prolate head and contractile tail (typical myoviral morphotype) was observed in the TEM images. According to morphological observations, information from the “Viral Zone” website, and guidelines from the International Committee on Virus Taxonomy (ICTV), the phage was suggested to belong to the class *Caudoviricetes* (formerly order *Caudovirales*) and myoviral morphotype (formerly family *Myoviridae).*

### Thermal stability and pH

The lytic spots vanished entirely at 70 °C where the phage lysate ceased its lytic activity. As a result, the thermal inactivation point was determined to be 70 °C. However, at pH 2–10, the phage lysate continued to exhibit its lytic activity with a marked decrease in lytic activity at pH 11–12.

### Genomic characterization of Salmonella_phage_VB_ST-SA173

**Fig. 4:**
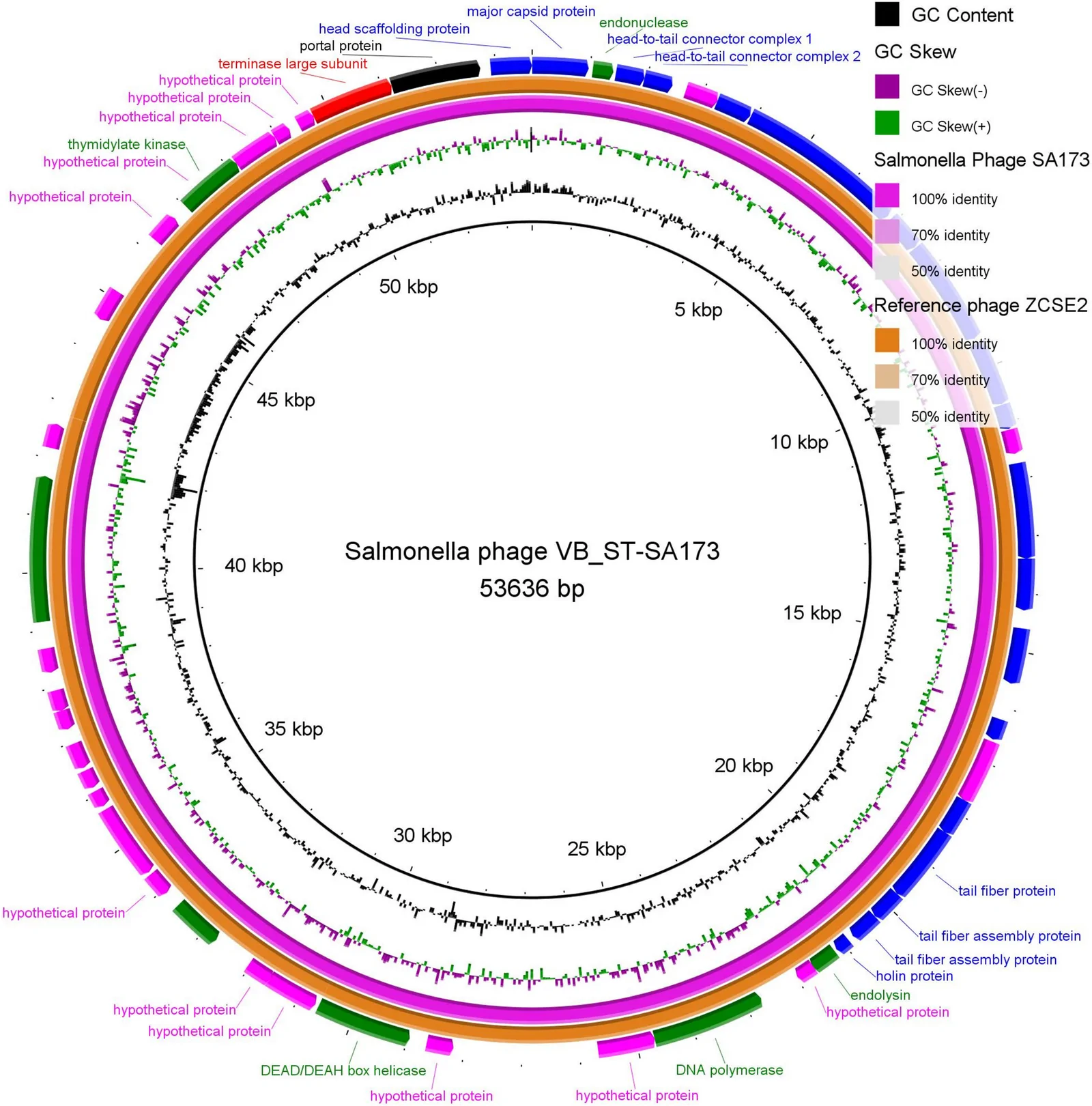
Circular genome map of Salmonella_phage_VB_ST-SA173*.* (NCBI GenBank Accession code PQ351918; size 53,636 bp, purple ring) and the reference phage (Salmonella_phage ZCSE2, complete genome; NCBI accession code NC_048179.1; size 53,965 bp, orange ring). The color coding of genes indicates the functional categories of putative proteins: structural proteins (blue), terminase protein (red), non-structural proteins (green), portal proteins (black), and hypothetical proteins (fuchsia). The creation of the circular image was performed using the BLAST Ring Image Generator (BRIG) tool v0.95 (https://sourceforge.net/projects/brig/, accessed on 19 September 2024)

Using the qualified R9.4.1 flow cells (FLO‐MIN106) apparatus, high-throughput sequencing of the lysate of Salmonella_phage_VB_ST-SA173 produced a consensus sequence of 53,636 bp containing 53 ORFs (36 coded by + frames and 17 coded by − frames). Table S2 displays the ORF analysis and feature annotations for the Salmonella_phage_VB_ST-SA173. This phage was taxonomically classified as *Viruses*, *Duplodnaviria*, *Heunggongvirae*, *Uroviricota*, *Caudoviricetes*, *Loughboroughvirus*, and unclassified *Loughboroughvirus* according to BLASTn alignment analysis. The Salmonella_phage_VB_ST-SA173 circular genomic map is shown in Fig. 4.

### In vitro evaluation of antibacterial activity and stability of phage-loaded hydrogel

The phage-loaded hydrogel and phage lysate alone (positive control) both exhibited a consistent positive spot test with a distinct lytic zone on the bacterial lawns. However, the control hydrogel (negative control) showed no bactericidal activity (Fig. S1). Additionally, the phage-loaded hydrogel retained its lytic activity for a month after storage at 4 °C.

### In vivo activity of the phage-loaded hydrogel against CRAB* Acinetobacter baumannii*

#### The survival rate% of animal groups and photographs of wounded skin

**Fig. 5:**
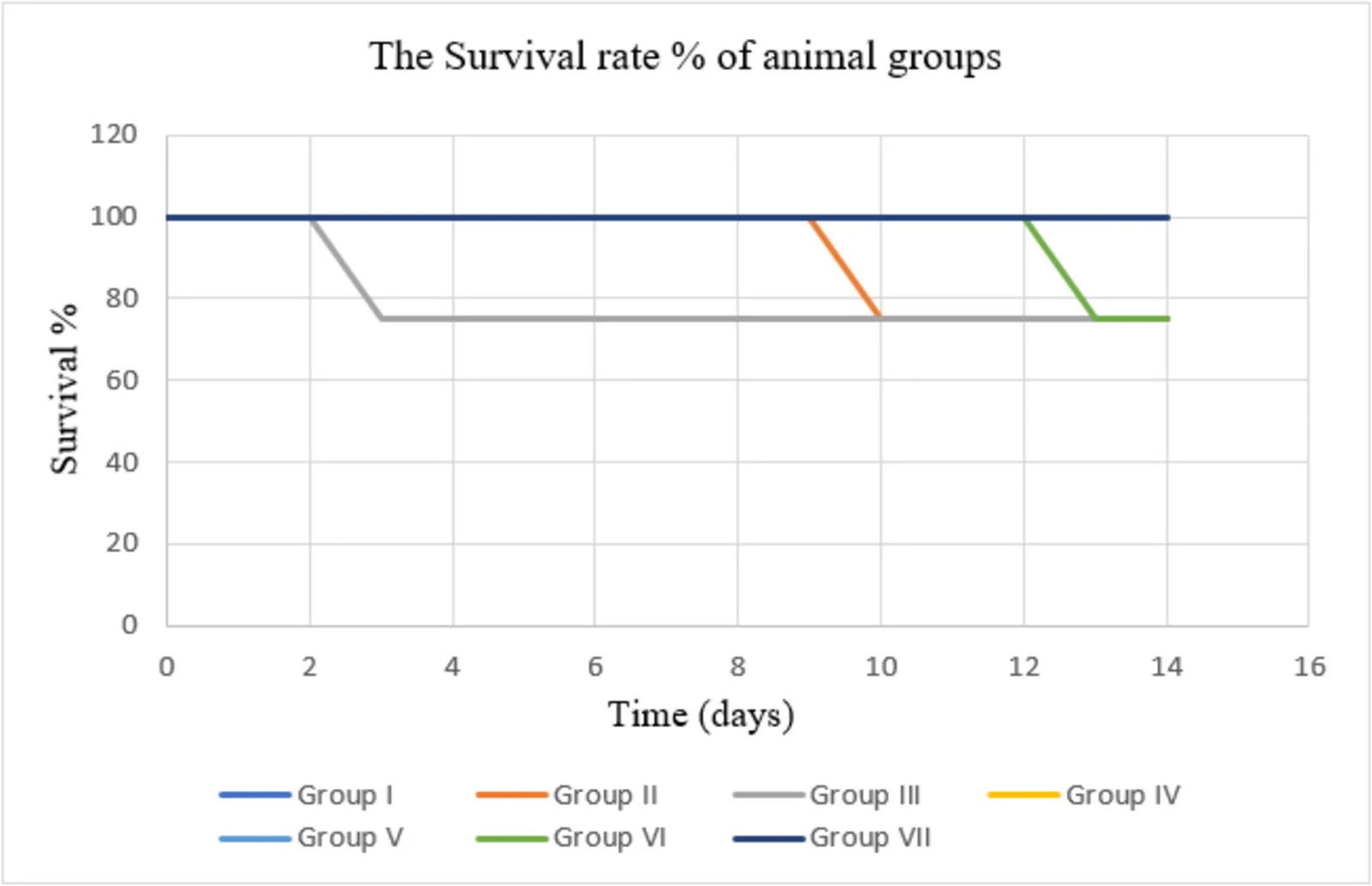
The survival rate percentage of the control animal groups and the treated animal group with *Salmonella* phage VB_ST-SA173–loaded hydrogel over 14 days

**Fig. 6:**
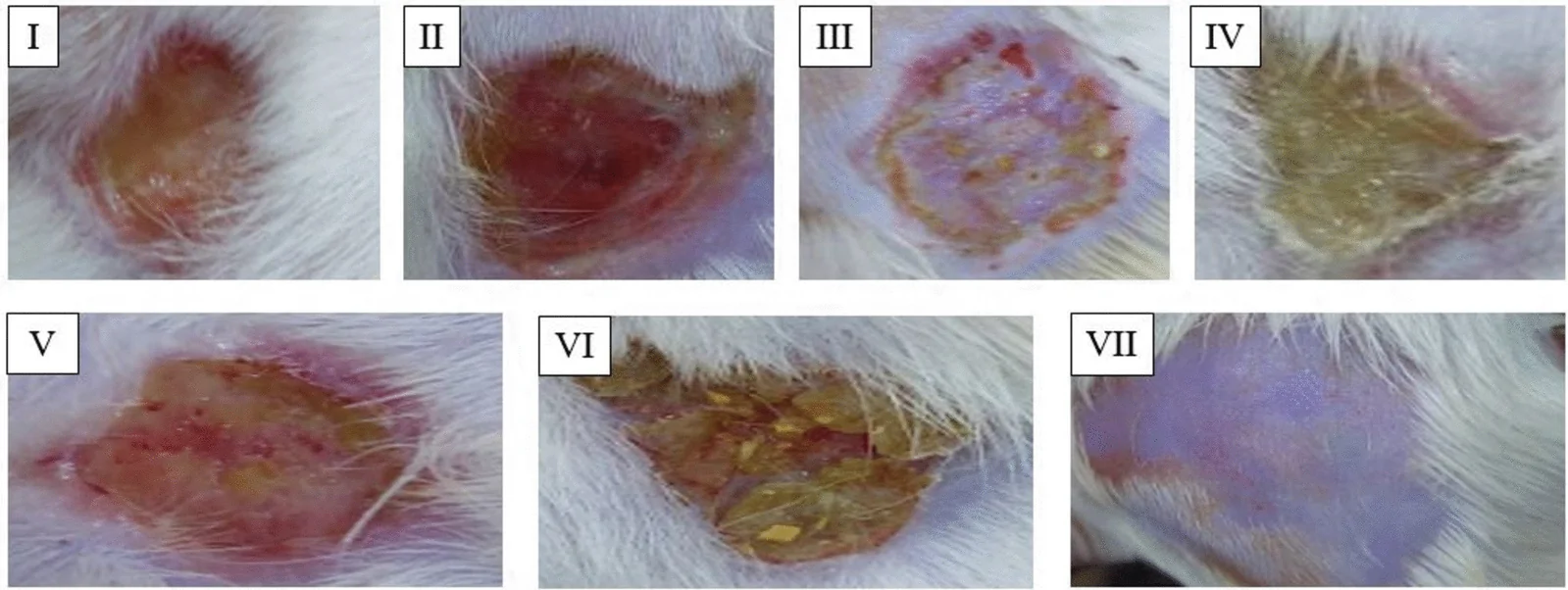
Photographs of wounded skin on the 14th day after injury in a representative rat from each group. Groups I, II, III, and VI demonstrated poor healing responses. Group IV showed a healing response to phage hydrogel treatment superior to the control groups. Group V had a better healing response than groups I, II, III, and VI. Group VII exhibited normal skin

After 14 days, the survival rate% for each group and the photographs of wounded skin after injury in a representative rat from each group are displayed in Figs. 5 and 6, respectively.

#### Histopathological examination

**Fig. 7:**
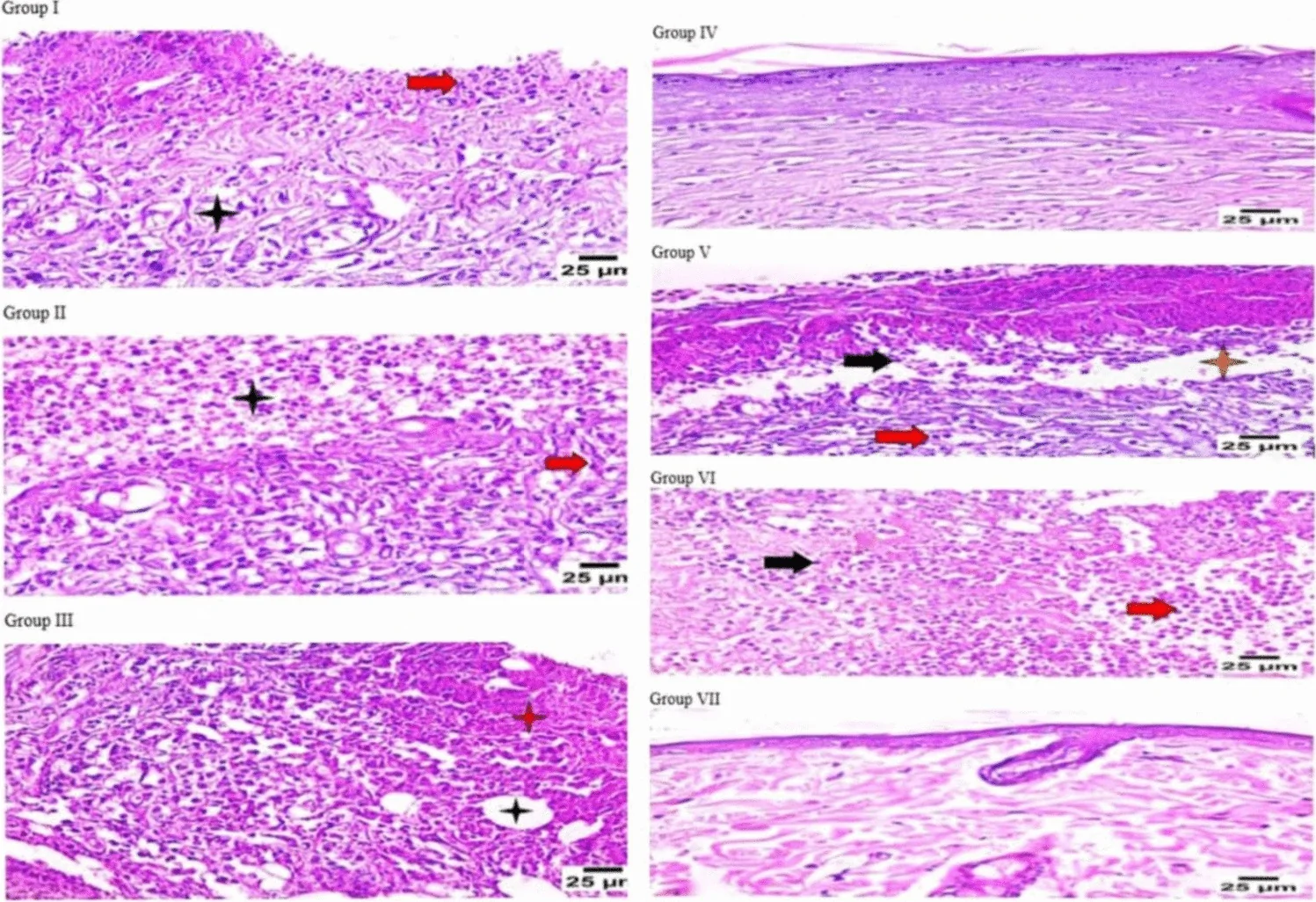
Microscopical examination of wound healing process in various groups and skin layers histopathological characteristics (H&E X 400). **I** Group I displayed coagulative necrosis, scattered collagen fibers (star), epidermal layer shedding, and inflammatory cell infiltration in the dermis, primarily lymphocytes and neutrophils (red arrow). **II** Group II revealed mild response to healing, polymorphonuclear cells infiltrating the top layer of the dermis (star), and necrotic tissue with large numbers of inflammatory and fibroblast cells (arrow) invading the dermis layer. **III** Group III showed poor healing response, necrotic tissue (red star), diffuse infiltration of inflammatory cells, primarily lymphocytes and neutrophils, vacuolation (black star), and the development of connective tissue in the dermal layer. **IV** Group IV showed apparent healthy skin with apparent normal epidermal layer and dermis. **V** Group V displayed a modest response to healing, epidermal layer separation (star) and inflammatory cells infiltrating the dermis, primarily neutrophils (black arrow) and lymphocytes (red arrow). **VI** Group VI displayed connective tissue formation in the dermal layer, dermal edema, diffuse necrotic tissue with significant infiltration of inflammatory cells, primarily neutrophils (red arrow) and lymphocytes (black arrow), and a moderate response to healing. **VII** Group VII exhibited normal skin structure of epidermal and dermal layers

After the examination of skin samples from various groups under a microscope, we found that:

Group I (control, burned, non-infected, untreated) displayed coagulative necrosis, scattered collagen fibers, epidermal layer shedding, and inflammatory cell infiltration in the dermis, primarily lymphocytes and neutrophils (Fig. 7I). Group II (control, burned, infected, untreated) revealed a mild response to healing, polymorphonuclear cells infiltrating the top layer of the dermis, and necrotic tissue with large numbers of inflammatory and fibroblast cells invading the dermis layer (Fig. 7II). Group III (control, burned, infected, treated with vehicle) showed a poor healing response, necrotic tissue, diffuse infiltration of inflammatory cells, primarily lymphocytes and neutrophils, vacuolation, and the development of connective tissue in the dermal layer (Fig. 7III). Alternatively, group IV (burned, infected, treated with tested hydrogel) showed apparent healthy skin with apparent normal epidermal and dermal layers (Fig. 7IV). Group V (positive control, burned, infected, treated with gentamycin 0.1%) displayed a modest response to healing, epidermal layer separation, and inflammatory cells infiltrating the dermis, primarily neutrophils and lymphocytes (Fig. 7V). Group VI (positive control, burned, infected, treated with beta-sitosterol) displayed connective tissue formation in the dermal layer, dermal edema, diffuse necrotic tissue with significant infiltration of inflammatory cells, primarily neutrophils and lymphocytes, and a moderate response to healing (Fig. 7VI). Group VII (normal control, intact, non-infected, untreated) exhibited normal skin structure of epidermal and dermal layers (Fig. 7VII).

## Discussion

In our study, we recovered two phages from environmental sewage. The *Salmonella*_phage_VB_ST-SA173 was chosen for further study and identification because it displayed completely clear inhibition zones on bacterial lawns as well as transparent plaques that confirmed the phage’s lytic effectiveness. The presence of plaques with identical morphology, size, and shape proves the existence of a pure virus, whereas the presence of plaques with varied features indicates the presence of many viruses (Bhat et al. 2022). In addition, the phage titer was estimated in terms of plaque-forming unit (PFU) per milliliters as previously reported (Abd-Allah et al. 2022). Furthermore, lytic (virulent) phages exhibit clear and transparent plaques, while temperate phages display opaque and turbid plaques (Park et al. 2020). Six CRAB isolates were susceptible to the lytic activity of the Salmonella_phage_VB_ST-SA173 (21.43%). The phages vB_AbaP_W8 and vB_AbaSt_W16, which belong to the *Autographiviridae* and *Straboviridae* families, respectively, showed lytic activity against 24.14% and 34.48% of CRAB strains. However, the vB_AbaSi_W9 phage, which has not been categorized, exhibited a broader lytic spectrum; however, only 10.34% of CRAB clinical isolates displayed clear lysis (Choi et al. 2024).

Interestingly, Salmonella_phage_VB_ST-SA173 also exhibited lytic activity against three MDR *Salmonella* spp. clinical isolates (60%). Similarly, SE7 and SE20 phages exhibited lytic activity against 62.30% and 57.38% of *Salmonella* spp*.* clinical isolates, respectively. Consequently, Salmonella_phage_VB_ST-SA173 was regarded as a polyvalent phage that showed lytic activity against strains of two different genera.

It is worth noting that the fundamental shortcoming of the previous phage taxonomy classification, morphology-based family, was an incorrect depiction of evolutionary history (Aiewsakun et al. 2018). The International Committee on Taxonomy of Viruses (ICTV) issued a new taxonomy release (#37), genome-based taxa, in 2022 (https://ictv.global/). The new genome-based taxonomy provided better knowledge of the diversity and genetic interactions among the numerous and different viruses. The morphology-based families *Myoviridae* (contractile tail), *Podoviridae* (short non-contractile tail), and *Siphoviridae* (long non-contractile tail) removal and the order *Caudovirales* substitution with the class *Caudoviricetes* were the most significant changes in the recent phage classification, where the *Caudoviricetes* class included all tailed bacterial and archaeal viruses having double-stranded DNA genomes and icosahedral capsids (Turner et al. 2023). The presence of a tailed phage with a characteristic prolate head and contractile tail was demonstrated by the TEM images of the Salmonella_phage_VB_ST-SA173. It is suggested that the phage belongs to the *Caudoviricete* class with a myoviral morphotype based on its morphological characteristics. Additionally, there are morphological similarities between Salmonella_phage_VB_ST-SA173 and other Salmonella phages that belong to the *Loughboroughvirus* genus.

The whole genome sequence of the *Salmonella*_phage_VB_ST-SA173 has been submitted to the NCBI GenBank database under accession code PQ351918. The genome of the phage comprises 53 ORFs and a 53,636 bp linear dsDNA molecule with 45.9% G + C content. The Salmonella_phage_VB_ST-SA173 is most similar to *Salmonella* phage ZCSE2 (identity, 99.06%; coverage, 100%), according to a comparison of the genome of the Salmonella_phage_VB_ST-SA173 with nucleotide sequences that were previously submitted to the NCBI database. The Salmonella_phage_VB_ST-SA173 and *Salmonella* phage ZCSE2 were taxonomically classified as *Viruses*, *Duplodnaviria*, *Heunggongvirae*, *Uroviricota*, *Caudoviricetes*, and* Loughboroughvirus*. The *Salmonella* phage ZCSE2 genome is a 53,965 bp linear dsDNA molecule with 78 putative ORFs and 45.83% G + C content (Mohamed et al. 2020). Interestingly, phage S144, which belongs to *Loughboroughvirus* genus, is a polyvalent phage that exhibits lytic activity against *Salmonella* spp. and *Cronobacter sakazakii*. It is fascinating to note that the *Loughboroughvirus* genus’s tail fiber C-terminus resembles the *Cronobacter* phages GAP31 and GAP32. Due to the crucial role of the C-terminus in the tail fiber in host recognition and binding, it is suggested that SE4 and ZCSE2 phages that belong to *Loughboroughvirus* genus may also infect *C. sakazakii* and act as polyvalent phages (Gambino et al. 2020). Additionally, *Klebsiella* phage VB_KpM-AEV23, a member of *Loughboroughvirus* genus, was isolated via *Klebsiella pneumoniae* bacterial isolate https://www.ncbi.nlm.nih.gov/nuccore/2642221666.

The Salmonella_phage_VB_ST-SA173 retained its lytic activity at pH 2–10, with a significant decrease in lytic activity at pH 11–12, indicating low stability in highly alkaline settings. Similarly, A. baumannii vB_AbaM_AB3P2, a new species in the *Obolenskvirus* genus, exhibited its lytic activity at pH 2–10 and was completely inactivated at pH 11–12 (Tan et al. 2024). Moreover, ZCSE2 phage maintained its lytic activity at pH 4–9 with a striking decrease in phage titer at pH 3, indicating low stability at extremely acidic settings (Mohamed et al. 2020). In terms of thermal stability, Salmonella_phage_VB_ST-SA173 maintained its lytic activity up to 60 °C before ultimately deactivating at 70 °C. Similarly, A. baumannii phages Ab_WF01, BUCT628, and pIsf-AB02 completely lost their lytic activity at 70 °C (Sisakhtpour et al. 2022) (Zhu et al. 2022) (Wang et al. 2024). Both the temperature and pH stability of Salmonella_phage_VB_ST-SA173 are beneficial for practical applications.

We prepared four distinct phage hydrogel formulations for our study, utilizing xanthan gum, Carbopol 940, agarose, and carboxymethyl cellulose. The most stable gel formulation that retained its viscosity and physical characteristics was Carbopol 940, which was subjected to autoclave-steam sterilization at 121 °C for 15 min. Our findings were consistent with those of the earlier investigation (Bindal et al. 2003). By spotting hydrogel formulations on bacterial lawns and then incubating them for 24 h at 37 °C, the release of infectious phages from the formulations was qualitatively evaluated (Brown et al. 2016).

The largest and clearest lytic zones were observed in Carbopol 940 phage hydrogel, suggesting an excellent phage release profile. Carbopol 940 hydrogel alone had no antibacterial action against the specified CRABa clinical isolate (Fig. S1). Furthermore, Carbopol 940 phage hydrogel maintained its lytic activity for 1 month, suggesting phage stability during the period of pre-clinical studies (14 days). For the pre-clinical assessment of topical phage-loaded hydrogel, Carbopol 940 phage hydrogel was selected.

The potential of the phage hydrogel to eradicate the bacterial infection was assessed using a thermal injury model in male rats infected with the CRABa strain to prevent female hormones from interfering with the healing process (Troncoso et al. 2020). The animal groups were divided into one test group and six control groups, each consisting of four rats. Histological analysis of several injured groups revealed various degrees of tissue damage and healing processes. Group I (control, burned, non-infected, untreated) was created to compare the damage caused by CRABa infection in this group to that of the other groups. Group II (control, burned, infected, untreated) was created to examine the virulence and pathogenicity of CRAB1, the degree of chronic inflammation that interferes with healing, and the efficacy of topical phages in the burn-wound infection model. Group III (control, burned, infected, treated with hydrogel without phage) was designed to validate the accuracy of the findings and prove the phage’s complete therapeutic activity. Group IV (burned, infected, treated with tested hydrogel) was employed to assess the phage-loaded hydrogel’s antibacterial activity and healing potential. To serve as standards for comparing antibacterial activity and wound healing, two positive control groups were created. In group V, the bacterial-infected wound was treated with gentamycin. In group VI, beta-sitosterol was used to treat the infected burn because of its wound-healing characteristics. Group VII (normal control, intact, non-infected, untreated) was created and examined. Our results showed enhanced survival in the burned-infected groups that were treated with phage hydrogel (group IV) and gentamycin (group V). Additionally, in comparison to the other treated groups, group IV showed a significant improvement in wound healing, as evidenced by apparent healthy skin with normal epidermal and dermal layers. Our results verified the efficiency of Salmonella_phage_VB_ST-SA173 in controlling wound-associated infections with CRAB clinical isolate.

In conclusion, in this research, a polyvalent Salmonella_phage_VB_ST-SA173 from an environmental sewage sample showing a lytic activity against CRAB and MDR *Salmonella* spp. has been identified, purified, and characterized. The TEM images showed a tailed phage with a characteristic prolate head and a contractile tail. The phage’s molecular analysis indicated that it belongs to *Loughboroughvirus* genus. Throughout a broad range of temperatures and pH levels, Salmonella_phage_VB_ST-SA173 maintained its stability and lytic activity. These features allow the phage to be applied in a variety of dosage forms and applications. When applied to burn wounds infected with CRAB, the hydrogel of the phage lysate demonstrated lytic activity and significantly accelerated wound healing. Salmonella_phage_VB_ST-SA173 can be a good candidate for the treatment of CRAB infections. Furthermore, the phage exhibited a promising lytic activity against MDR *Salmonella* spp. isolates.

## Supplementary Information

Below is the link to the electronic supplementary material.

Supplementary file1 (PDF 19.5 MB)

## Data Availability

The authors declared that the article and its supplementary file provide the data that supports the study's conclusions. The phage genome's final assembled sequence was annotated and submitted to the NCBI GenBank database with the accession code PQ351918.
