# Supplementary material for: Genomic characterization and pre-clinical evaluation of a new polyvalent lytic Loughborough phage
Source: Appl Microbiol Biotechnol. 2025 Aug 2;109(1):177. doi: 10.1007/s00253-025-13559-2 (PMC12317907; doi:10.1007/s00253-025-13559-2)
Supplement: Supplementary file 1 — Supplementary file1 (PDF 19.5 MB) [file 253_2025_13559_MOESM1_ESM.pdf]

Applied Microbiology and Biotechnology

Genomic characterization and pre-clinical evaluation of a new polyvalent lytic *Loughborough* phage

Mahmoud M. Sherif[1], Neveen A. Abdelaziz[1], Sarra E. Saleh[2], Khaled M. Aboshanab*[2]

[1]Department of Microbiology and Immunology, Faculty of Pharmacy, Ahram Canadian University, Sixth of October City, Giza 12451, Egypt

[2]Department of Microbiology and Immunology, Faculty of Pharmacy, Ain Shams University, Cairo 11566, Egypt

*** Correspondence: Prof. Dr. Khaled M. Aboshanab**

Microbiology and Immunology Department, Faculty of Pharmacy, Ain Shams University, Cairo 11566, Egypt

Tel: +202-24051120, Fax: +202-24051107

Email: **aboshanab2012@pharma.asu.edu.eg**

ORCID: **https://orcid.org/0000-0002-7608-850X**

Table S1 Phenotypic and genotypic analysis of the used CRAB clinical isolates.

Isolate code	Susceptibility pattern					MIC of IPM (µg/mL)	Beta-lactamase genes
	CL	IPM	DOX	AMK	LEV		
CRABa	S	R	R	R	R	128	bla_{NDM}, bla_{VIM}
CRABb	S	R	R	R	R	512	bla_{VIM}
CRABc	S	R	R	R	R	512	--------------
CRABd	S	R	S	R	R	16	bla_{oxa-23}
CRABe	S	R	R	R	R	64	bla_{oxa-23}
CRABf	S	R	R	R	R	64	bla_{oxa-23}, bla_{PER-1}

CL, colistin; IPM, imipenem; DOX, doxycycline; AMK, amikacin; LEV, levofloxacin; MIC, minimum inhibitory concentration; bla_{PER-1}: *Pseudomonas aeruginosa* Extended spectrum RND-1 (group A Beta lactamase); $bla_{NDM:}$ New Delhi metallo-beta-lactamase (group B beta-lactamase); $bla_{VIM:}$ Verona integrin associated metallo-beta-lactamase (group B beta-lactamase); $bla_{oxa-23:}$ Oxacillinase beta lactamase (group D beta-lactamase).

Table S2. *Salmonella* phage VB_ST-SA173 feature annotations and open reading frame (ORF) analysis

ORF Number	Feature ORF name	Strand	Interval range
1	major capsid protein	+	1..978
2	HNH endonuclease	+	1051..1410
3	putative head-to-tail connector complex 1	+	1456..1956
4	putative head-to-tail connector complex 2	+	1956..2456
5	hypothetical protein	+	2702..3277
6	tail fiber protein	+	3277..3897
7	tail protein	+	3912..6854
8	structural protein with Ig domain	+	6924..7565
9	major tail protein	+	7578..9452
10	tail sheath	+	9540..10679
11	virion structural protein	+	10690..11121
12	hypothetical protein	+	11138..11566
13	tail length tape measure protein	+	11732..13393
14	tail fiber protei	+	13390..14286
15	Phage protein (ACLAME 213)	+	14599..15570
16	baseplate spike	+	15560..16204

ORF Number	Feature ORF name	Strand	Interval range
17	baseplate wedge subunit	+	16213..16584
18	hypothetical protein	+	16624..17751
19	structural protein	+	17744..18397
20	tail fiber protein	+	18390..19739
21	tail fiber assembly protein	+	19739..20281
22	tail fiber assembly protein	+	20284..20793
23	Holing	+	20895..21158
24	endolysin	+	21202..21678
25	hypothetical protein	+	21657..21986
26	DNA polymerase	-	22712 ... 24682
27	hypothetical protein	-	24679 ... 25677
28	hypothetical protein	-	28183… 28653
29	DEAD/DEAH box helicase	-	28956 ... 30629
30	Cas4-domain exonuclease	-	30692 ... 31603
31	hypothetical protein	-	31569 ... 32033
32	hypothetical protein	-	32094 ... 32633
33	Sak4-like ssDNA annealing protein	-	32738…33628

ORF Number	Feature ORF name	Strand	Interval range
34	hypothetical protein	-	33918 ... 34343
35	Phage protein	-	34390 … 35709
36	hypothetical protein	-	35776 ... 36072
37	hypothetical protein	-	36141 … 36467
38	hypothetical protein	-	36519... 36971
39	hypothetical protein	-	37239… 37580
40	hypothetical protein	-	37577... 37945
41	hypothetical protein	-	38262 … 38687
42	DNA primase	+	39153….41681
43	Phage protein	+	42176….42604
44	hypothetical protein	-	44561 ... 45127
45	hypothetical protein	+	46230….46739
46	hypothetical protein	+	47019….47669
47	thymidylate kinase	+	47666….48166
48	hypothetical protein	+	48163….48966
49	hypothetical protein	+	48966….49253
50	hypothetical protein	+	49430….49726

ORF Number	Feature ORF name	Strand	Interval range
51	terminase large subunit	+	49726….51174
52	portal protein	+	51176….52738
53	head scaffolding protein	+	52917….53633

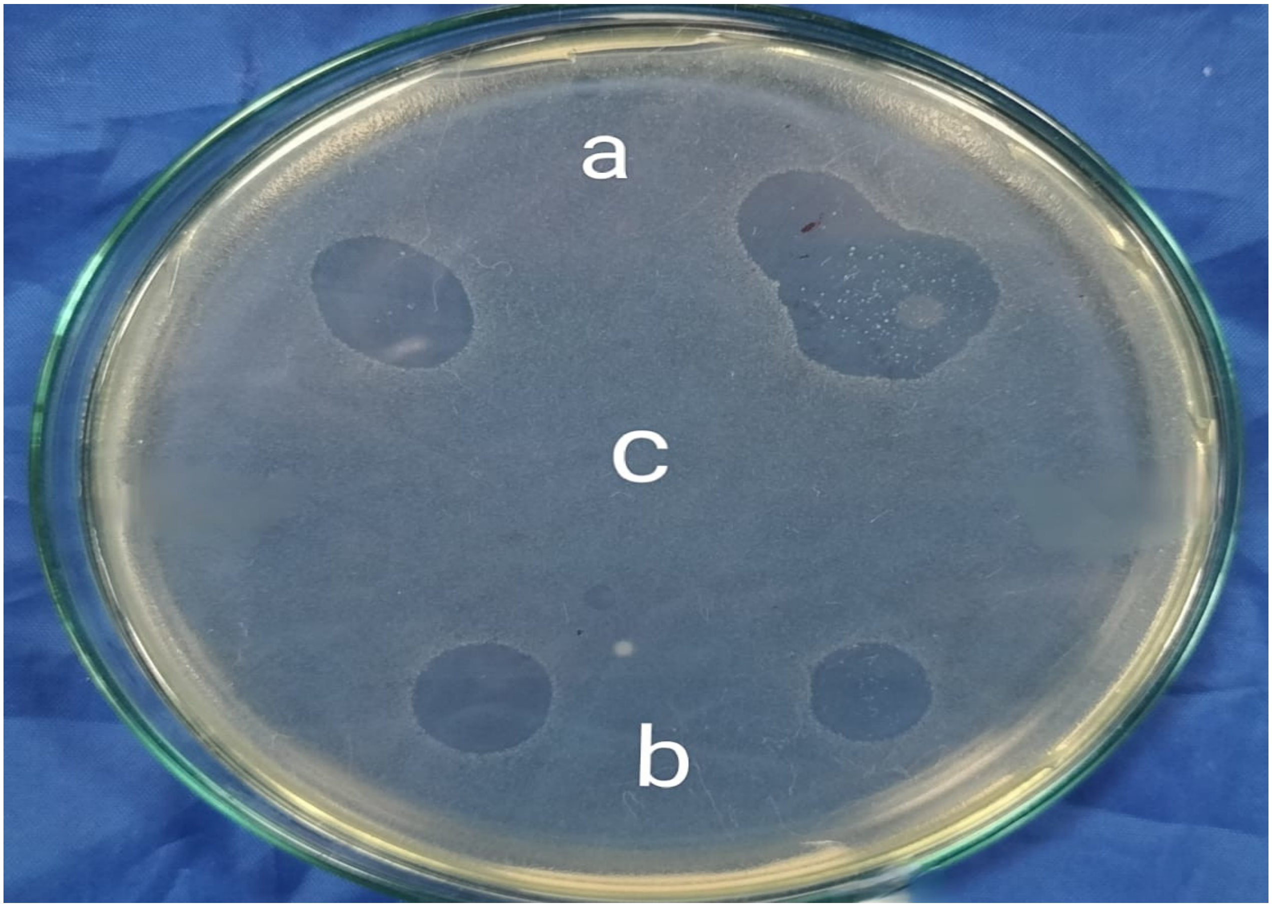

Fig.S1 In vitro activity of the phage-loaded hydrogel against carbapenem-resistant *Acinetobacter baumann*ii **(a)** Clear lytic zones were observed with phage lysate alone, **(b)** Similarly, Clear lytic zones were observed with phage-loaded hydrogel, **(c)** The hydrogel by itself exhibited no antibacterial effect.

.

Original uncropped file

Group I

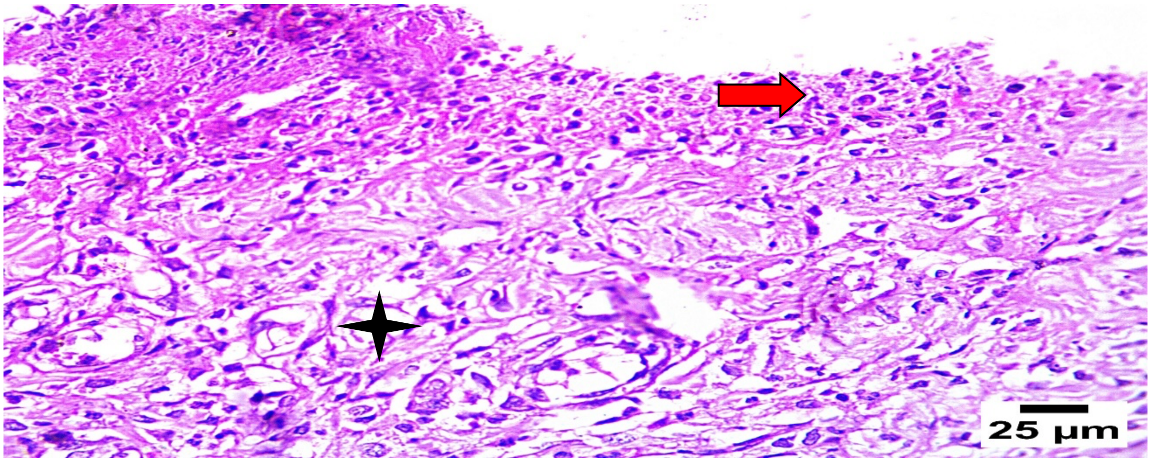

Group II

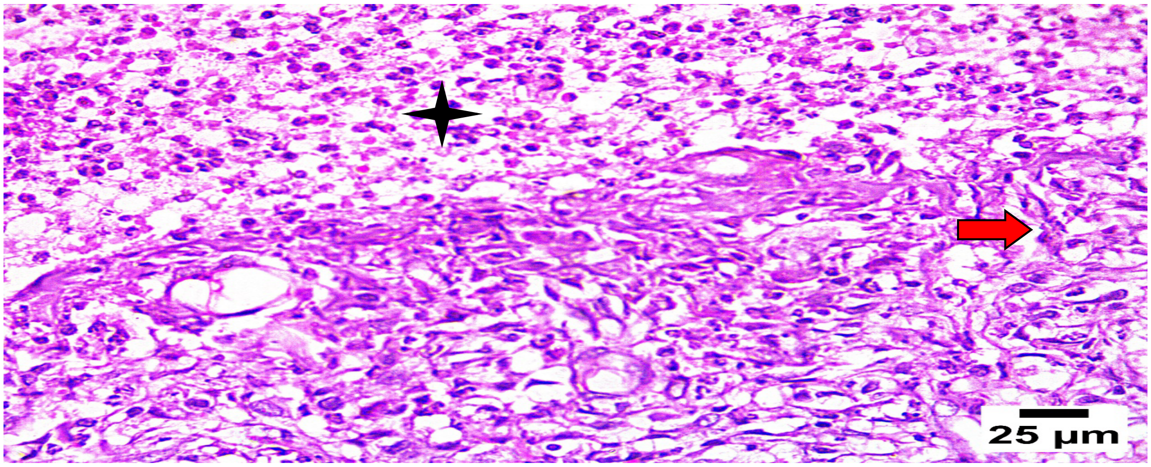

Group III

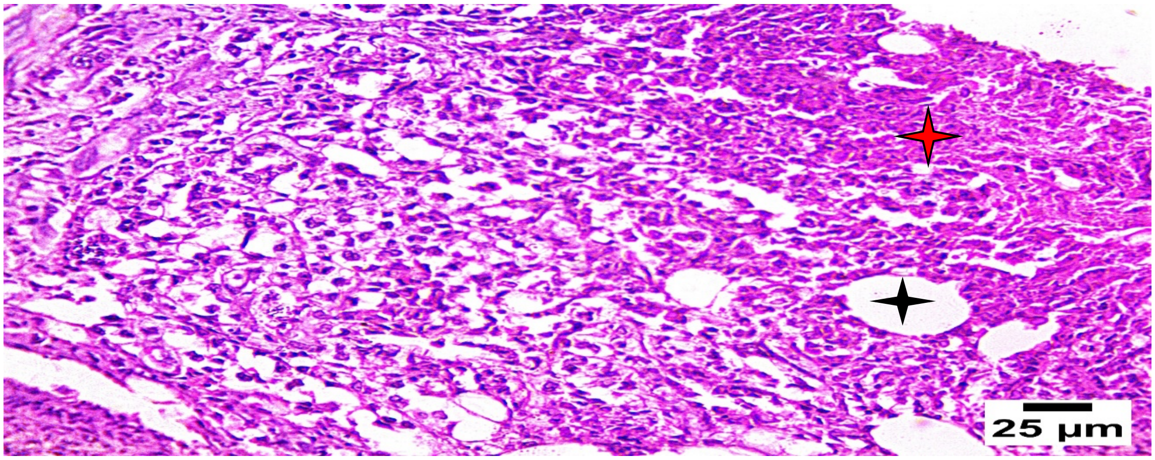

Group IV

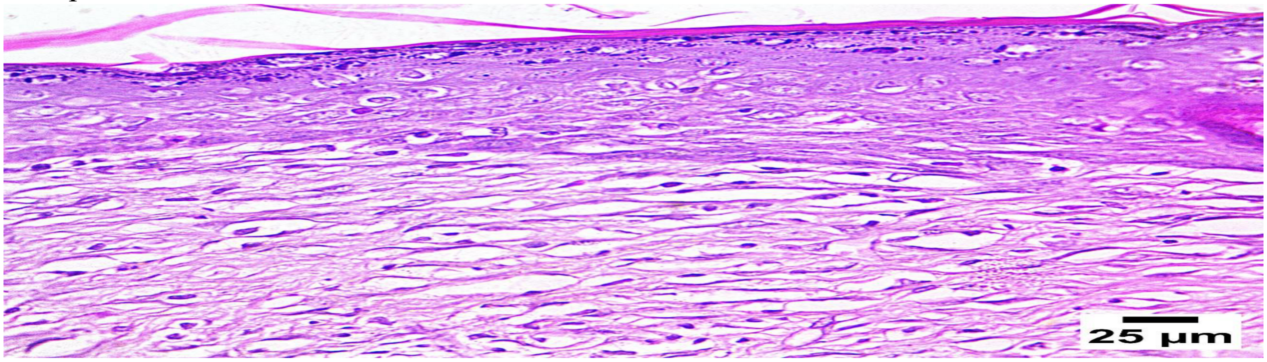

Group V

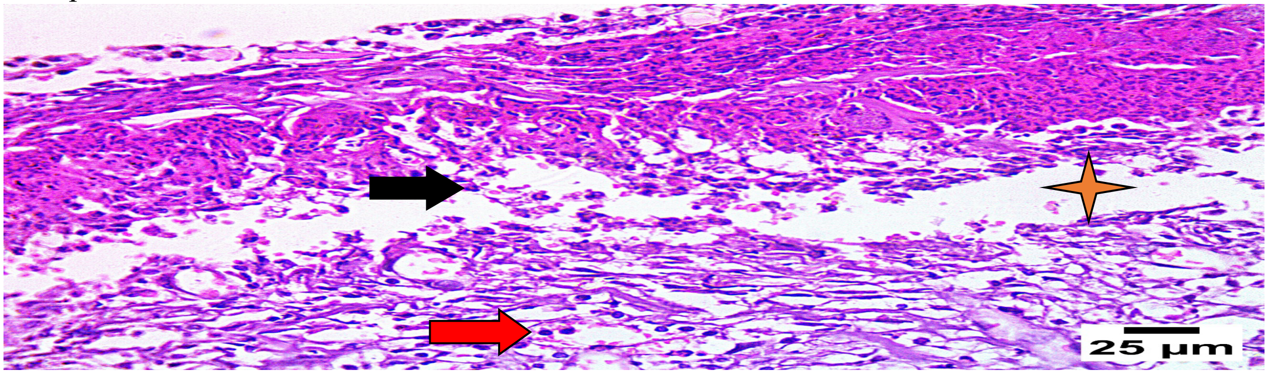

Group VI

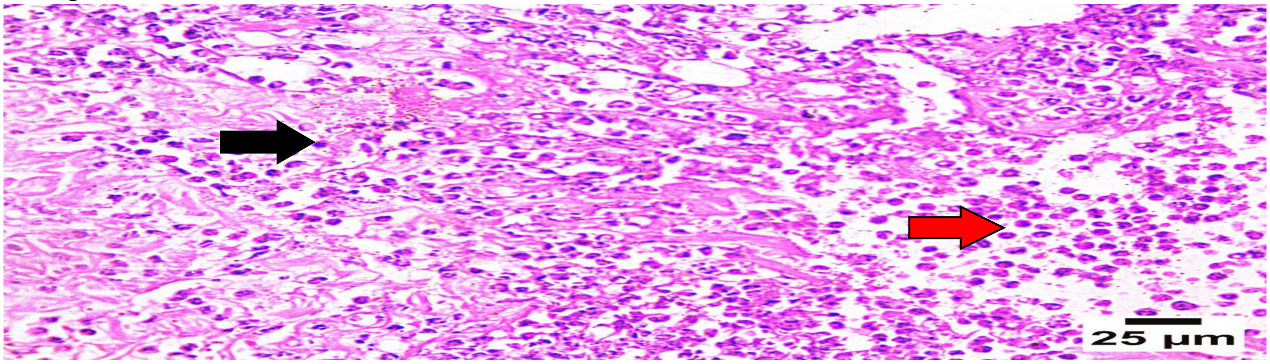

Group VII

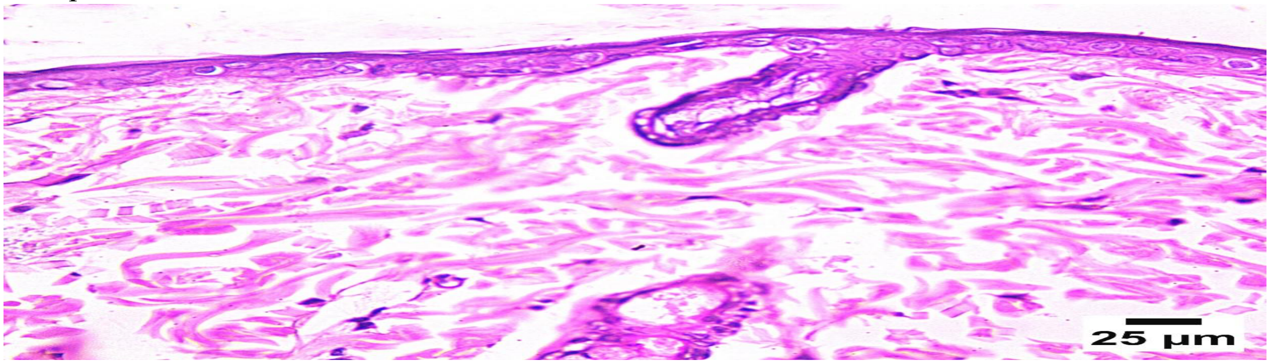

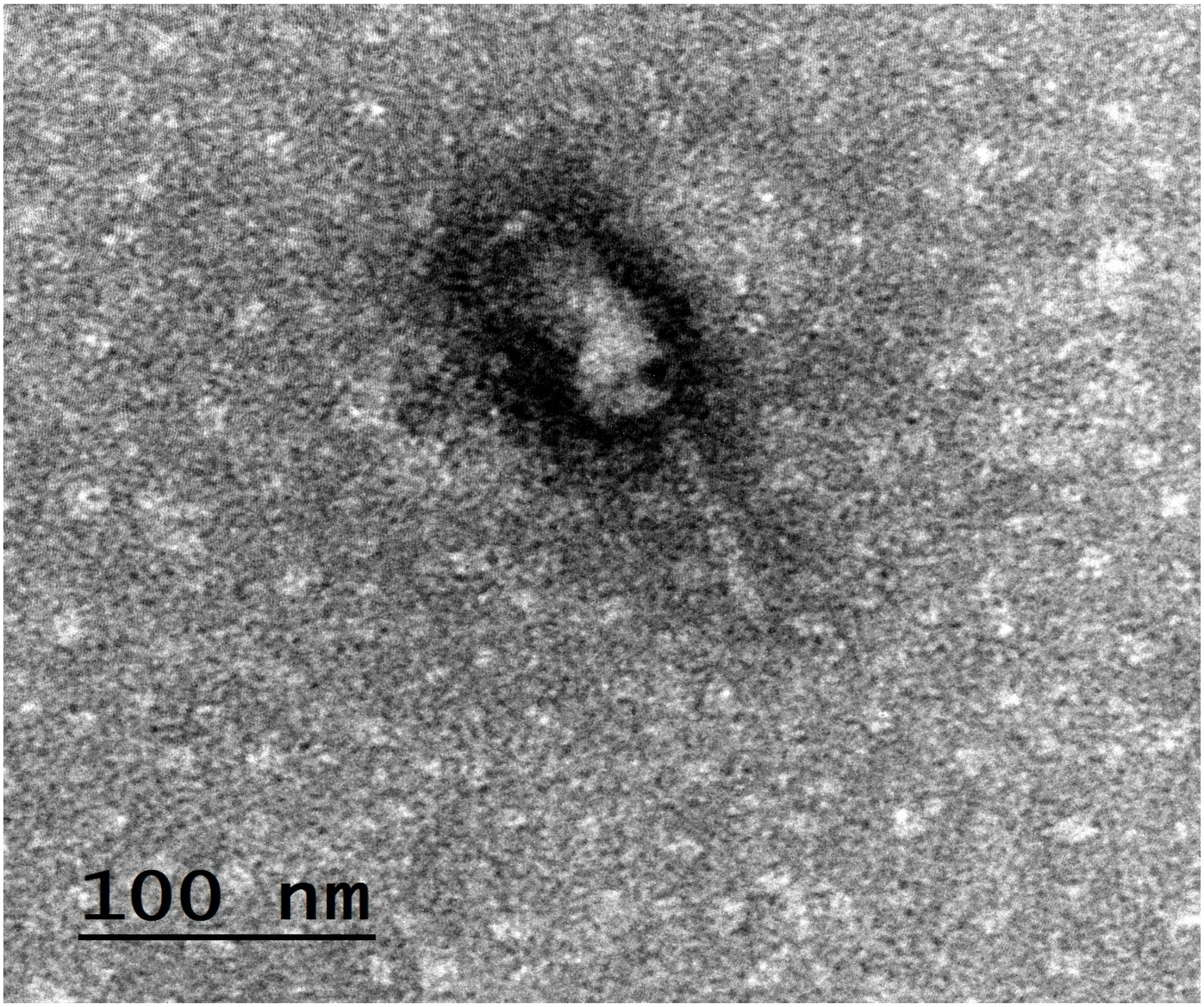
100 nm
